# The pharmaceutical applications of palm kernel oil: innovations in dermatological therapies

**DOI:** 10.3389/jpps.2026.16205

**Published:** 2026-10-05

**Authors:** Teong Gee Thor, Rana Sejare, Xin Yi Teoh, Ahmed Bassam Farhan, Sri Fatmawati, Siok Yee Chan

**Affiliations:** 1 School of Pharmaceutical Sciences, Universiti Sains Malaysia, Penang, Malaysia; 2 School of Pharmacy, Monash University Malaysia, Subang Jaya, Selangor Darul Ehsan, Malaysia; 3 Pharmaceutics, College of Pharmacy, Baghdad University, Baghdad, Iraq; 4 Department of Chemistry, Faculty of Science and Data Analytics, Institut Teknologi Sepuluh Nopember, Surabaya, Indonesia; 5 Indonesian Herbal and Horticultural Science and Technology Park, Del Institute of Technology, Humbang Hasundutan, North Sumatera, Indonesia

**Keywords:** antibacterial, dermatological, moisturiser, palm kernel oil, skincare

## Abstract

Dermatological problems of the skin, hair or nails are considered one of the most cutaneous complaints and form around 30% of all visits to dermatologists. Recent treatments have been focused on exploring natural agents as alternatives for antibiotic-free treatments. Palm kernel oil (PKO) is identified as one of the healthy alternatives and is incorporated into traditional healthcare as a result of its safety and efficacy on the human body. PKO is a vegetable oil which is extracted from the seeds of the palm plant fruit. It has various therapeutic benefits, especially on skin health including antibacterial, antifungal, emollient, moisturiser, antioxidant and anti-ageing effects. Moreover, the moisturising potential can prevent dryness and enhance the elasticity of the skin. These remarkable effects could be the key to encouraging the pharmaceutical industries and researchers to further utilise and incorporate pharmacological properties of PKO in developing natural pharmaceutical skincare and dermatological products as a promising safe, sustainable and cost-effective alternative. This article highlights the important pharmacological effects and the latest pharmaceutical applications of PKO and its derivatives.

## Introduction

Dermatological problems of skin, hair or nails are considered one of the most prevalent cutaneous complaints which constitute 30% of all visits to dermatologists [1] It is characterized typically by papules, pustules and skin nodules, in addition to the psychological and emotional interests which have negative impact on human life [2]. The most common skin diseases include acne, eczema, and atopic dermatitis, which are associated with microbial involvement from gram-positive bacteria such as *Propionibacterium acnes, Staphylococcus epidermidis,* and *Staphylococcus aureus,* as well as the gram-negative bacterium *Escherichia coli* [3]. Other common dermatological conditions include inflammatory disorders such as rosacea and psoriasis, hair-loss conditions or also known as alopecia, and parasitic infestations of the hair and scalp [4].

The earliest management of dermatological disorders emphasised the effective role of lifestyle behaviours such as diet, sleep and exercise to treat skin disorders such as acne and psoriasis [4]. The strategies to treat dermatological disorders are based on the intensity of the disease which ranges from mild, moderate to severe [5]. The mild cases can be treated using antiseptics, topical antibiotics or corticosteroids [6]. In more severe cases, oral antibiotics are used alone or in combination with topical or systemic corticosteroids [7].

During the last decade, many people who live in tropical countries have been using plant oils such as coconut and palm oils in their lives for dermatological cosmetics products as well as traditional health care [8, 9]. These plant oils are characterised by their safety and compatibility, as well as their therapeutic effects [10] owing to the saturated and unsaturated fatty acids [11]. A study determined the effectiveness and safety of virgin coconut oil compared with mineral oil as a therapeutic moisturizer for mild to moderate xerosis which is considered one of the common skin conditions characterized by dry, rough, scaly, and itchy skin associated with a defect in skin barrier function and treated with moisturizers. This study was applied on a randomized double-blind controlled clinical trial was conducted on mild to moderate xerosis in 34 patients with negative patch-test reactions to the test products [12]. The study was concluded that coconut oil is as effective and safe as mineral oil when used as a moisturizer [12].

Some studies compared the benefits and properties of these oils based on the composition of fatty acids [13, 14]. Among the studied plant oils, flaxseed oil, soybean oil and palm oil (PO) which are categorized as polyunsaturated fats, have shown poor oxidative stability [15]. Monounsaturated fats, such as olive oil, are comparatively less susceptible to oxidation due to their single double bond structure. In this regard, palm kernel oil (PKO) exhibits greater oxidative stability because it is rich in saturated fats, particularly lauric and myristic acids, and contains a low content of unsaturated fatty acids, resulting in fewer double bonds susceptible to oxidation. [14, 16, 17]. Moreover, therapeutic benefits of PKO on the skin and hair have been reported [18]. Given the cost-effectiveness and potential of PKO for use across various industries, particularly in topical skin products [19], a critical review of the scientific findings and recent developments on its usage is warranted. This narrative review therefore aims to highlight the key pharmacological effects and latest pharmaceutical applications of PKO and its derivatives.

## Methodology

A structured, three-phase approach was adopted for this review: (i) planning and scoping the search strategy, (ii) systematic collection of relevant articles, and (iii) analysis and comparison of findings across the identified literature. Google scholar, PubMed were used as the primary database for peer-reviewed journal articles published from 2000 to 2026. Patent literature was searched separately using Lens.org^1^.

The following keywords, used individually and in combination, guided the search: “palm kernel oil”, “Elaeis guineensis,” “PKO,” “derma,” “medical” and “dermatology”. Combinations (e.g., “palm kernel oil” AND “dermatology”; “PKO” AND “derma”) were applied to capture literature spanning traditional use, physicochemical and therapeutic properties, and pharmaceutical applications of PKO, with particular emphasis on skin and hair preparations.

## Development and distribution

The development of PKO began gradually after 1850 in Africa on a farm that contained oil palm trees [20]. During 1850 and 1970 PKO changed from a locally traded African resource into a major global agricultural and industrial product [21]. By 1970 and early 1980, Malaysia and Indonesia became one of the leading producers and exporters of PO and PKO, accounting for 85% of the world’s total production [22, 23]. Ever since, scientists have explored the importance and benefits of this oil. In 1984, PKO was taken into consideration dramatically in comparison with other plant oils, such as coconut oil. Tang and Teoh showed that PKO is a highly saturated oil with a lower free fatty acid content, making it suitable for use as a raw material in soap manufacturing [24]. Additionally, Chen and Berger compared the use of PKO, other fatty acids and tallow in soap manufacturing. The findings of the study revealed that PKO reduced skin problems such as irritation owing to its lower content of octanoic and decanoic fatty acids [25]. Furthermore, another study conducted by Ooi and Pee indicated that PKO requires no extra treatment prior to use, thereby offering advantages in manufacturing applications [26].

In 1979, the Palm Oil Research Institute of Malaysia (PORIM) was responsible for palm oil development and finding new refineries. PORIM has now been referred as the Malaysian Palm Oil Board (MPOB), it started the industrial master plan (IMP) in the year 1985–1996 to shift its attention from pure palm oil production to developing new refineries of crude PKO oleochemicals, especially PKO. The Board addressed the applications of PKO as an alternative to cocoa butter. After 1996, MPOB set up the second IMP, which considered PKO a pharmaceutical fatty acid emulsifier [27].

## Composition of PKO

Palm Kernel Oil is extracted from the seeds of the *Elaeis guineensis* palm fruit [28] as shown in Figure 1.

**FIGURE 1:**
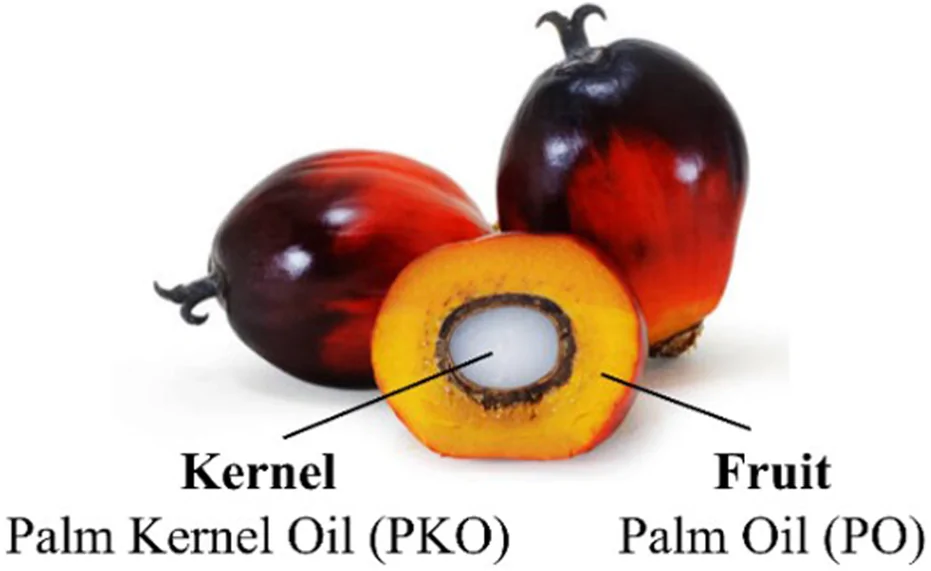
Elaeis guineensis fruit [28].

Comparison of the compositions between PKO and PO are shown in Table 1. These differences created diversity in their applications based on the desired purposes. Both oils consist of triglycerides and a combination of glycerol and different fatty acids [31].

**TABLE 1:** The composition’s differences between PKO and PO.

Composition	Palm kernel oil	Palm oil	References
Fatty acids	Highest ratio of saturated fatty acids	40% monounsaturated fatty acids 10% polyunsaturated fatty acids saturated fatty acids form 50%	[29]
Lauric acid C_12:0_	45.61%	0.18%	[30]
Myristic acid C_14:0_	16.26)%	0.86%	
Oleic acid C_18:1_	17.14%	41.59%	
Palmitic acid C _16:0_	9.70%	42.24%	
Caprylic acid C_8:0_	2.85%	0.0`%	
Linoleic acid C_18:2_	2.78%	10.72%	
Others	5.57%	4.41	​

Unlike PO, PKO contains a mixture of medium fatty acids including lauric acid (C_12_) that forms the highest component, followed by myristic acid (C_14_) [32]. Figure 2 represents the structure of lauric and myristic acids. These fatty acids have rendered PKO’s suitability to act as a solid lipophilic carrier which can deliver and protect lipophilic compounds more effectively than a liquid carrier [33]. Furthermore, PKO fatty acids such as lauric acid exert therapeutic effects of on the human body [34]. While, PO has a higher concentration of long chain of unsaturated fatty acids mainly C16:0 [30].

**FIGURE 2:**
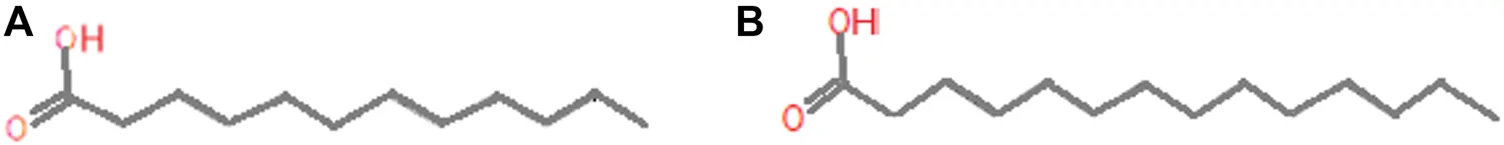
Structure of PKO fatty acids **(A)** Lauric acid, **(B)** Myristic acid (produced by ChemDraw software).

On the other hand, PKO contains higher amount of β-sitosterol (65–73%) as compared to crude PO (56–59%). In addition, PKO has a greater β-tocopherol content compared to PO. While, tocotrienols which are responsible for the antioxidant activity and may decrease the free radicals, are present at the lowest concentration in PKO [35]. The composition of PKO fatty acids can differ depending on the botanical source and processing conditions. In particular, differences in cultivar type, growing environment, and harvesting practices may influence the concentration of medium-chain fatty acids, especially lauric and myristic acids, which are the major constituents of PKO [36].

Additionally, the steps of industrial processing such as extraction method can alter the overall fatty acids composition [37]. These variations may affect its physicochemical properties, stability and performance in pharmaceutical and cosmeceutical applications. In this context, Niamketchi Gilles Léonce et al, investigated the physicochemical properties and antifungal activity of PKO extracted from the palm kernels accessions of traditional palm trees [38]. These accessions were collected from five different regions across the city of Man in Ivory Coast, including Blole, Dompleu, Douele, Gbantongouin, and Gbangbegouine, where all palm fruit kernels were processed through the same processing conditions before the extraction. The findings of this study revealed that there were variations in the oil content, fatty acid composition, physicochemical and functional properties depending on the collected area of palm trees. The oil yields exhibited a range from 50.72 to 55.55%, with the Blole and Dompleu regions demonstrating the highest yields of 55–56%. The oxidation index of PKO from Gbantogouin showed a lower level of lipid degradation with the highest levels of peroxide value 5.07 meq O_2_/kg. Additionally, the acid value of the five PKO samples was found to be in accordance with the Codex standard of 4 mg KOH/g of oil. The PKO from Douele exhibited higher saponification index (245.85 mg KOH/g of oil). Furthermore, the antifungal activity of these types of PKO against *Aspergillus sp* was slightly different based on the collected area [38]. Hence, high quality oil with bioactive and functional properties is associated to the source of oil.

PKO can also be transformed into derivatives such as esters (PKOEs), glycosides and mannosides. PKOEs are manufactured by transesterification technique using the lipase enzyme as a catalyst and oleyl alcohol as the reactant. The resulting PKO esters contain a mixture of esterified fatty acids including 54.1% oleyl laurate ester that forms the highest component, 13.9% oleyl myristate ester, 6.4% oleyl oleate, 6.2% oleyl palmitate. Esterified fatty acids have several advantages more than unesterified fatty acids such as improving stability of fatty acid against the oxidation and more solubility which can easily incorporate into the pharmaceutical formulations [39]. When PKOEs contain a higher ratio of medium-chain esters, they behave as moistening agents in semi-solid preparations [40, 41]. The dynamic role of these esters was evaluated in many studies to determine their properties and applications. For example, Martinez-Felipe et al. developed PKO glycosides by manufacturing PKO mannosides which showed a strong effect as a non-ionic surfactant, thereby allowing its effective application in cosmeceutical preparations [33]. Owing to this, these esters have been incorporated into pharmaceutical formulations as a great wetting agent with no oily texture. Therefore, this review attempts to shed light on the findings of the recent literature that focused on the important and updated pharmacological effects, as well as the latest pharmaceutical and cosmeceutical applications of PKO and its fatty acids.

## Physicochemical parameters of PKO and its derivatives

The need to use effective non-toxic and inexpensive oils or fatty acids may encourage the researchers to evaluate the potential of PKO that plays a valuable role in enhancing the stability of product during the production of pharmaceutical formulations, storage, suitability and safety after use [42]. As a result, it is crucial to determine the behaviour of oil within the formulation by measuring a few parameters of oil used in its characterization, quality and stability [42], including but not limited to (1) saponification value (SV) represents an indicator that is inversely proportional to the average molecular weight of the constituent fatty acids [43]. (2) Acid Level (AV) which refers to the degradation of oil ascribed to the free fatty acid (FFA) level. This value is essential in determining the stability upon storage. Third, iodine value (IV) which refers to the degree of unsaturation based on the number of double bonds in the oil [28]. As the oxidation rate of oil has a high impact on its stability and shelf life, it is considered a significant character to measure the oxidation or rancidity level of oil by calculating the peroxide value (PV) which expresses the reactive oxygen [44].

It was found that the IV of PKO was low (15.86 ± 4.02 mg of KOH/g) due to the abundance of saturated fatty acids [45]. Another study conducted by Bahadi et al demonstrated that the lower iodine value of PKO was owing to its composition of unsaturated fatty acids being low [46]. It was observed that SV was high at 232.815 mg of KOH/g. The presence of high amounts of short-chain fatty acids led to higher SV, and this was an advantage in soap production [47]. Moreover, the PV of PKO was calculated using the amount of active oxygen per 1 kg of oil. PKO has a PV of 1.70 meq/kg, but it was low compared to PO, which led to prolonging the storage time of PKO [47]. Finally, it was reported that AV of PKO was low (2.7 ± 0.3 mg of KOH/g) compared to coconut oil (3.9 ± 0.4 mg of KOH/g) due to the lower enzymatic hydrolysis of glycerides in PKO, causing less free fatty acid formation which leads to better storage quality and lower degree of rancidity [45]. They discovered that PKO also contains some minerals such as potassium, calcium, and sodium [47]. Another group by Martinez-Felipe et al. concluded that the high concentrations of sodium and potassium in the PKO can hinder high blood pressure [33].

The IV of PKO esters is higher than its oil because of the interaction between oleyl alcohol and triglyceride of PKO to form esters. As a result, the high IV improved the moistening effect. It was found that the melting point of esters is lower than the oil because the esters have lower molecular weights [31, 48]. This feature assisted the researchers in incorporating PKOEs into nano and microemulsions [49], where the esters melt rapidly and entirely during the preparation of emulsion without applying high temperatures for melting. Furthermore, fatty acid esters have been successfully integrated into topical and transdermal formulations, including low melting point esterified saturated fatty acids such as oleyl laurate ester, oleyl myristate ester, oleyl oleate and oleyl palmitate [41, 50]. As a result, formulations can be applied and softened at body temperature quickly, while decreasing the potential for degradation of the active ingredients and improving the thermal stability of the emulsions.

Atasie and Akinhanmi investigated PKO’s physical characteristics and values of PKO to get its suitability for human consumption and its ability to be used in the industrial field [47]. The melting point of PKO was recorded as 29 °C, slightly higher as compared to coconut oil (26 °C) [32]. This feature allows its incorporation into pharmaceutical formulations as emulsifying agents to stabilise an emulsion, decrease crystallization of the drug, enhance drug solubility and improve drug permeability and bioavailability, consequently longer drug shelf life [51–53].

In summary, PKO is generally considered safe for topical applications when it is adequately refined. However, regulatory requirements differ across regions, especially regarding acceptable levels of free fatty acids, peroxide value, and residual processing contaminants [54]. Accordingly, stability assessment is critical to ensure PKO safe and effective use in final products.

## Therapeutic effects of PKO

Many studies have shown the benefits of PKO on the human body health specially its dermatological effects on the skin [16, 34, 55, 56]. Dermato-therapeutics effects of PKO which include moisturising, antibacterial and antifungal, antioxidant and anti-ageing effects, are further detailed in the subsection below.

### Antibacterial effect (wound healing)

The antibacterial activity of PKO was evaluated by Ubgogu et al, where authors revealed that the antibacterial activity coincides with the amount of lauric acid in PKO [57]. Ubgogu studied different samples of PKO which had different concentrations of lauric acid based on the sources and methods of preparation of various palm kernel oils. The inhibitory effect of PKO which contained higher ratio of lauric acid (9.3%) on *Staphylococcus aureus* was the highest, followed by *Streptococcus*. Although the PKO shows limited antibacterial activity against these types of bacteria, it may still help prevent or delay the emergence of bacterial resistance. This literature concluded that PKO with high lauric acid concentration has the potential to be applied on the skin and reduce bacterial infections. An additional study conducted by Loung et al. displayed a similar antibacterial activity of virgin coconut oil and hydrolysed PKO against *Streptococcus aureus*, *Salmonella thypi* and *E. coli*. The results exhibited that PKO had higher inhibitory effect against gram-positive bacteria (*Staphylococcus aureus*) than the effect against gram-negative bacteria such as *S. thypi* and *E. coli* [58]. Another study demonstrated the potential of lauric acid as a natural antibiotic^2^ against *P. acnes* for acne treatment, which promotes follicular inflammation (inflammatory acne). This study evaluated the antimicrobial property of lauric acid against *P. acnes* both *in vitro* and *in vivo*, where lauric acid effectively decreased the number of *P. acnes* colonized with mouse ears, thereby relieving *P. acnes*-induced ear swelling and granulomatous inflammation^3^ [3]. The study conducted by Kabara showed that lauric acid is the most inhibitory saturated fatty acid against gram-positive organisms [59].

Auttajinda et al studied the antibacterial efficacy of PKO fatty acids: lauric acid and palmitic acid against *Staphylococcus aureus* and *E. coli* at different ratios. The results displayed that the presence of lauric acid in the fatty acids combination enhanced the antibacterial activity of palmitic acid [60]. Furthermore, Nainggolan and Sinaga confirmed the antibacterial activity of PKO resulting from lauric acid against gram-positive bacteria. In the human body, lauric acid is transformed to monolaurin, which is responsible for the antibacterial effect. This modification protects the skin from the bacterial infection [30].

The antibacterial effect of PKO and its fractions has been associated with wound-healing activity, as Zulkifli and co-workers demonstrated the significant efficacy of PKO esters as a wound-healing agent [61]. The researcher has successfully produced polyurethane (PU) from PKOEs to be applicable as a dressing for highly drainage surface wounds. *Staphylococcus aureus* and *E. coli* were suppressed for 24 and 48 h [62]. Ibrahim et al. also demonstrated the wound-healing effects of PKO as compared to the expensive commercial topical wound-healing products, such as 1% silver sulfadiazine and 5% povidone-iodine antibacterial creams [63]. In addition, PKO activity was also compared with the cheapest conventional treatments such as shea butter and honey. The results showed that the superior and prompt wound healing efficacy was noted for PKO and povidone-iodine after 6 days of treatment (p < 0.05) based on the rapid reduction in the wound size and on the eighth day, the wound in both treatments recovered completely.

### Antifungal effect

The antifungal activity of PKO was evidenced through several research studies. Kamga et al. evaluated the inhibitory activity of virgin coconut oil and PKO on six types of *Candida* species [64]. The data exhibited that the susceptibility of PKO toward fungi was higher than coconut oil. *Candida lipolytica* was the most affected by the PKO inhibition effect compared to other species. Besides, the activity of antifungal drugs, such as fluconazole and nystatin on *Candida parapsilosis*, was enhanced in the presence of PKO. In addition, it was found that PKO from Blole and Dompleu regions of Man city in Ivory Coast had greatest antifungal properties. This effect was observed within 24 h after the addition of 75 µL of PKO to plates containing Aspergillus sp., with an inhibition rate reaching 72% [38] All the above researchers attributed the inhibitory effect on fungi and antifungal activity to the presence of lauric acid in PKO [56].

### Emollient and moisturizing effects of PKO

Emollients and moisturizing creams are used to break the dry skin cycle and to maintain the smoothness of the skin. Dryness is frequently related to an impaired barrier function observed, for example, in atopic skin, psoriasis, ichthyosis, and contact dermatitis [65]. A study conducted by Chiabi et al. evaluated the moisturising and emollient effects of PKO for skin softening. The results displayed the effective role of PKO on sensitive newborn skin with decreased skin peeling and dermatitis by forming a lipid film barrier amongst the corneocytes. The lipid film holds to the upper layer of the epidermis (stratum corneum) and thus prevents water evaporation. Consequently, it moisturises the skin, avoids dryness and enhances the elasticity of the skin [66]. Another study carried out by Viviane et al. showed the emollient activity of PKO in cosmetic and pharmaceutical industries due to the presence of lauric acid [55]. Recently, Adebisi and Sosanya revealed the remarkable role of PKO as an effective emollient and lubricant for dry skin due to its high contents of fatty acids [34].

### Antioxidant and antiaging effects

PKO has noteworthy fatty acids and antioxidant compounds which are able to reduce the signs of ageing by different mechanisms. Krist et al. reported a high antioxidant potential of PKO compared to PO due to the higher content of β-Tocopherol in PKO [35]. A 3. blished work by Asagba et al. confirmed the antioxidant and anti-ageing properties of medium and short-chain fatty acids-containing PKO [67]. Additionally, Wong et al. investigated the antioxidant effect of PKO [68], this study showed antioxidant activity was associated with the presence of phenolic compounds in the oil. In addition, it was noticed that the antioxidant activity increased significantly with an increase in the contents of total phenolic compounds rather than total flavonoid compounds. Young and Godwin compared the presence of different fatty acids composition of PKO as well as vegetable oils like olive and groundnut oils and their effects on human skin health [69]. A similar effective organic compound, myristic acid, which is the second highest compound of PKO was reported. Additionally, palmitic acid, stearic acid, 9- octadecenol 2,3-epoxycarane and oleic acid were detected in PKO. These compounds retard ageing, stimulate repairing of the injured lipid cells and protect the skin from ultraviolet (UV) radiation.

## Skin and hair care products

The development and incorporation of PKO into the beauty industries was a result of the notable effects of the oil on the skin and human health [70]. Many cosmetic companies have shifted to produce green products by incorporating natural-origin materials into their formulations. Coconut oil, PKO and its fractions have shown an excellent consideration as emulsifying agents in cosmetics and skin products, such as cream, ointment and lotion [31]. Mahadi et al. utilised PKOEs as an oily phase to formulate *Phyllanthus urinaria* ethanolic extract-loaded PKOEs nanoemulsion as an anti-ageing skin localised cream. This study found that the optimised skin nanoemulsion showed the potential to cross through the skin layers and form a barrier, thereby protecting against the dangerous effects of UV rays. This protection was achieved by inhibiting the free radicals and reactive oxygen species. Accordingly, it could be applicable in cosmeceutical products such as anti-ageing and sunscreen skin creams. These effects may be attributed to the properties of PKO which can perform as a shield against UV light [71].

In 2012, Aripin et al conducted another study to compare PKO glycosides and PO glycosides as cutaneous carriers to encapsulate dl-α-tocopherol (vitamin E) in unilamellar vesicles [29]. The data of this study exhibited the capability of PKO glycosides to encapsulate vitamin E for dermal delivery compared to PO glycosides. Moreover, it was noted that a low concentration of PKO lipids significantly enhanced the encapsulation performance of the vesicles, improved the storage stability and increased the shelf life of the cosmetics skin products. Furthermore, Che Sulaiman et al. incorporated PKOEs as an oily phase into the nanoemulsion of *Clinacanthus nutans* extract. PKOEs were combined with guava seed oil (GSO) in a ratio (9:1) to enhance the absorption of the extract. The optimised nanoemulsion successfully penetrated the skin layers and delivered the extract as an anti-ageing agent with non-irritating properties. Thus, the study exhibited that the presence of PKO glycosides enhanced the stability of formulation for 3 months at accelerated conditions: 40 ± 2 °C/75 ± 5% RH [72].

In terms of hair care, a study conducted by Tuo-Kouassi et al evaluated the properties of coconut oil and PKO to formulate shampoos for black hair. The findings of the study revealed that the presence of lauric acid, had a good affinity of these oils to hair proteins, which were able to penetrate the cuticle and cortex of the hair, resulting from their low molecular weight (<1,000 Da). Moreover, linoleic acid is believed to enhance hair growth in the scalp and eyebrows as well [73].

### Commercial/market products

Owing to the effective wound-healing properties of PKO, some companies have formulated this oil with other excipients or alone as a wound healer product. The pharmaceutical company (Kernel fresh) incorporated PKO with glycerin without adding preservatives to be used as a natural wound healing product for external application. Claims of the product include to mend the damaged skin and nourish dry hair due to its vitamin E contents. Recently, this company has produced two beauty products of PKO in combination with shea butter: cream and soap which are one of the facial beauty secrets [74].

In addition, Botanical Beauty company produced organic PKO as a regenerative skin and hair product. PKO has been significantly considered as a topical agent to heal the skin rapidly [75].

African Naturalistas company has manufactured a product that contains 100% pure PKO to treat skin and hair problems resulting from the features of PKO and its derivatives [76]. The company claims that it can strengthen, nourish, moisturise and smooth dry skin and hair rapidly. Commercially, it also marketed as being able to treat skin peeling and enhance hair growth due to the presence of antioxidants like vitamin E. In one of the patented documents, it was claimed that PKO and its derivatives is an alternative to cocoa butter, published on 03-08-1999 from Ohio US09/050,938^4^ [77]. In 2006, an invention DE112006004214B4 [78] was granted that has been associated with the antimicrobial properties of modified PKO with a high concentration of MCT such as lauric acid and fatty acids similar to coconut oil. Some companies reported the nourishing, hydrating and moisturizing effects of PKO on the skin and hair [74–76].

## Pharmaceutical application as lipid carrier excipient

The evolution of pharmaceutical and cosmetics industries expands the research interest to evaluate the potential of PKO and its fatty acids in several oleo-chemical fields due to PKO’s nature of non-toxic and inexpensive nature [32, 79]. Research has also proven the significant role of PKO fatty acid esters as excellent carriers in drug delivery systems comparable to vegetable oily carriers such as soybean oil, sunflower oil, and castor oil, which are used in lipid-based pharmaceutical formulations to improve the solubility and enhance bioavailability of poorly water soluble drugs [80, 81].

A study conducted by Cheikhyoussef and Cheikhyoussef exhibited the significant role of PKO in the pharmaceutical industry and drug delivery system due to its safety and non-toxic effects [82]. In the aspect of formulation, incompatibility of PKO was reported due to its high saturated fatty acid profile, which can contribute to issues such as crystallization, phase separation, or decreased stability when blended with certain unsaturated oils, emulsifying agents, or active pharmaceutical ingredients [83]. These physicochemical incompatibilities can influence product consistency, bioavailability, and overall shelf-life, particularly in dermo-cosmetic formulations. However, the selection of effective emulsifiers, homogenization to reduce the droplet size, and adjusting the formulation parameters such as viscosity, temperature, and pH may improve the emulsification of oils and reduce separation, thereby enhancing the long-term stability of the formulation [84, 85].

PKO and its derivatives have been used to replace jojoba and coconut oil as lipid carriers in nanoemulsion systems to deliver therapeutic drugs via topical and transdermal routes of administration, in addition to enhancing the solubility, permeability and bioavailability of drugs [86, 87]. Table 2 represents the application of PKO as lipid-based carriers.

**TABLE 2:** The application of PKO as lipid-based carriers.

Drug	Emulsion size/type	Surfactant	Administration route	Application	Ref.
Ibuprofen	16.52 ± 0.0816 nm O/W emulsion	Tween 80	Topical	Anti-inflammatory, analgesic effect	[88]
Ibuprofen	97.26 nm, O/W emulsion	Tween 80	Transdermal	Anti-inflammatory, analgesic effect	[50]
Hydrocortisone	O/W nanoemulsion	Tween 20/Lipoid S75	Transdermal	Steroidal Anti-inflammatory	[19]
Diclofenac sodium	79.85 nm, O/W nanoemulsion	Lecithin Cremophor	Transdermal	Anti-inflammatory, analgesic effect	[49]
CoQ10 enzyme	2.79–5.83 μm O/W emulsion	Sodium stearoyl lactate (SSL) SSL/k- carrageenan	Oral/topical	Nutraceutical and anti-ageing skin products	[89]

In 2012, the encapsulation of ibuprofen-loaded PKOE nanoemulsion was carried out by Salim et al. The study proved that the incorporation of hydrocolloid gums such as gellan and xanthan into the formulation considerably enhanced skin permeability of ibuprofen. The permeability increased more than four times in comparison to the traditional formula [88]. After that, Shahidan et al. effectively prepared the conventional transdermal ibuprofen loaded (PKOEs) nanoemulsion using two methods of emulsification homogenisation methods. The study demonstrated that the optimised formulation had good storage stability at room temperature. However, other cited effects such as the anti-inflammatory and antibacterial of ibuprofen have not been detected [50].

Several studies have reported the transdermal application of PKOEs. For example, Da Costa et al. developed a nanoemulsion formulation of hydrocortisone by using PKOEs as oily carrier. These esters have potential wetting and moisturising behavior without the greasy sensation after the skin application. Non-toxic solvents (such as ethanol and isopropanol) were added to the formulation to increase the stability of hydrocortisone in the nanoemulsion, while the solubility of hydrocortisone was improved by the presence of PKOEs. It was noticed that adding the solvent did not have any impact on the pH and particle size. Da Costa et al. further suggested applying this stable new formulation as a transdermal drug delivery carrier for hydrocortisone [19]. Another piece of literature conducted by Rezaee employed the central response surface methodology to design and characterise the impacts of four variables (amount of oil, surfactant, emulsifier, and water phase) on the viscosity and particle size. The anti-inflammatory drug, diclofenac sodium was formulated into a nanoemulsion for transdermal application using PKOEs as the lipid carriers. This optimised transdermal formulation showed privileges more than traditional oral diclofenac. For example, the gastrointestinal tract side effects were prevented and the first-pass metabolism of diclofenac was diminished. The developed formula also displayed distinguished stability during the storage period and long half-life [49].

## Discussion

Although palm kernel oil (PKO) has potential dermatological applications, its comedogenic potential warrants consideration in view of its high lauric-acid content. In the rabbit-ear model, Fulton [90] reported that medium-chain fatty acids were more potent than shorter- or longer-chain fatty acids in inducing follicular keratosis. More recently, Choi et al. [91] found that use of facial cleansers containing ingredients classified as comedogenic was independently associated with acne, while Starzyk et al. [92] emphasized that animal and isolated-ingredient assays may not reliably predict the comedogenicity of finished formulations in human skin. Collectively, these findings support a potential concern for lauric-acid-rich oils but cannot be directly extrapolated to native PKO, in which fatty acids occur predominantly esterified within triglycerides. Although lauric acid exhibits antimicrobial activity against Propionibacterium acnes [3], this does not preclude potential comedogenicity because antimicrobial activity and follicular comedogenesis are mechanistically distinct.

Available safety assessments indicate low sensitization potential for PKO at tested concentrations [93]. Nevertheless, allergic contact dermatitis has been reported with coconut- or lauric-fatty-acid-derived surfactants, including cocamidopropyl betaine. Recent patch testing in patients with cocamidopropyl betaine-related dermatitis demonstrated reactivity to manufacturing-related intermediates or impurities, including dimethylaminopropylamine and lauramidopropyl dimethylamine [94]. Thus, sensitization associated with chemically modified derivatives should not be attributed directly to native PKO. Further human studies of standardized PKO preparations and finished topical formulations are required to define their comedogenic and sensitization profiles.

### Sustainability and future prospect

Despite the great potential of PKO in dermatological therapies, its usage has been associated to several sustainability concerns. This is associated with the broader palm oil industry production where concerns, such as deforestation, loss of biodiversity and significant greenhouse gas emissions, especially when it is obtained from uncertified sources, have often been raised. Vijay et al. reported substantial regional variation in forest conversion associated with oil-palm development and identified biodiversity-rich forests vulnerable to future expansion [95] A global initiative of the Roundtable on Sustainable Palm Oil (RSPO) certification has been introduced to ensure sustainable cultivation of oil palm and production of palm oil/oil palm products, which impacts across people, planet and prosperity [96]. For people, this certification standard ensures fair labor practice, stakeholder equity and Free, Prior and Informed Consent (FPIC) for communities. In terms of the sustainability of the planet and prosperity, no deforestation, no planting on peat, protection of high conservation value forests and traceable supply chains were required for RSPO certification. The implementation of RSPO, especially in responsible sourcing by large multinational companies, was evidenced and published in their official platforms [97, 98]. On the other hand, the No Deforestation, No Peat, No Exploitation (NDPE) policy of palm oil supply currently serves as a strong implementation among industrials [99–101].

Nevertheless, certification should not be regarded as definitive proof of sustainability. Evidence concerning RSPO effectiveness remains mixed. Carlson et al. found that certification was associated with a reduction in deforestation in Indonesian plantations [102]. Conversely, tree-cover loss and habitat degradation have been documented within some certified concessions, indicating that certification alone does not guarantee the prevention of deforestation or biodiversity loss [103].

As the leading producers of PO and PKO, Indonesia and Malaysia have implemented strong centralised sustainability governance. In Indonesia, a mandatory Indonesian Sustainable Palm Oil (ISPO) certification has been enforced for all growers across the country [104]. The ISPO initiative aims to be the world’s largest sustainability scheme, where social and environmental responsibilities were included. Recognizing smallholders also play crucial role in the production of sustainable palm oil, the government of Indonesia provided financial support to smallholders to become ISPO certified according to the national standard [105]. This initiative was supported under the United Nations Development Program (UNDP) through its Sustainable Palm Oil (SPO) initiative. Similarly, in Malaysia, Malaysian Sustainable Palm Oil (MSPO) certification scheme was introduced and made mandatory as national framework for long-term sustainability [106]. Being recognized internationally, MSPO has been listed in the Standards Map of the International Trade Centre (ITC) [107]. On top of that, in 2025, European Union (EU) acknowledged the MSPO certification as a credible sustainability standard compliant to the EU Deforestation Regulation (EUDR) [108].

Overall, RSPO, ISPO, MSPO and NDPE frameworks are important but imperfect sustainability instruments. For dermatological PKO products, certified and traceable sourcing should be encouraged while recognising that certification alone cannot eliminate environmental and social risks. Consumers are encouraged to select transparently sourced and sustainability-certified oil palm products.

## Conclusion

The dermatological problems of the skin, hair or nails such as acne, eczema, atopic dermatitis and noticeable positive impact of PKO are presented in this review, it is worth mentioning that the exploration of use of PKO in dermal product may be of interest as a safe and low-priced alternative. PKO had the capability of acting as an antibacterial agent against *E. coli* and *Staphylococcus aureus* and repairing the wounds effectively. In addition to its crucial role as an emollient and moisturiser, it controls dead skin cell exfoliation and softening the skin, as well as restoring elasticity and flexibility, thereby recovering dry skin. These remarkable effects support the application of PKO in topical preparations to treat skin problems and complications resulting from acne and eczema such as dryness, irritation and bacterial growth. Hence, it is recommended to expand the research that assesses and elaborates on the moisturising and emollient influences of PKO, subsequently promoting its usage as an emollient in skin care products, especially for newborn babies and cosmetics preparations. Ultimately, the selection of sustainably-certified oils palm products with transparent sourcing plays a critical role in their quality, safety, pharmacological effects and pharmaceutical applications.

## Footnotes

1
http://lens.org/

2
https://www.sciencedirect.com/topics/pharmacology-toxicology-and-pharmaceutical-science/antibiotics

3
https://www.sciencedirect.com/topics/pharmacology-toxicology-and-pharmaceutical-science/granulomatous-inflammation

4
https://patents.google.com/patent/US5932275A/en?q=palm+kernel+oil&oq=palm+kernel++oil
